# Synthesis and Characterization of TiO_2_ Nanotubes (TiO_2_-NTs) with Ag Silver Nanoparticles (Ag-NPs): Photocatalytic Performance for Wastewater Treatment under Visible Light

**DOI:** 10.3390/ma15041463

**Published:** 2022-02-16

**Authors:** Achraf Amir Assadi, Sarra Karoui, Khaled Trabelsi, Anouar Hajjaji, Walid Elfalleh, Achraf Ghorbal, Mounir Maghzaoui, Aymen Amin Assadi

**Affiliations:** 1 Research Unit Advanced Materials, Applied Mechanics, Innovative Processes and Environment, Higher Institute of Applied Sciences and Technology of Gabes (ISSAT), University of Gabes, Gabes 6072, Tunisia; sarra.karoui20@gmail.com (S.K.); achraf.ghorbal.issat@gmail.com (A.G.); 2 Industries Chimiques du Fluor—Gabes Plant, 06 Rue Amine El Abbassi, Tunis 1002, Tunisia; m.maghzaoui@icf.ind.tn; 3 Industrial Zone Gabes Port, Gabes 6071, Tunisia; 4 Laboratoire de Photovoltaïque, Centre de Recherches et des Technologies de l’Energie, Technopole de Borj-Cédria, BP 95, Hammam-Lif 2050, Tunisia; khaled0984@hotmail.com (K.T.); physicshajjaji@gmail.com (A.H.); 5 Energy, Water, Environment and Process Laboratory, (LR18ES35), National Engineering School of Gabes, University of Gabes, Gabes 6072, Tunisia; walid.elfalleh@fst.rnu.tn; 6 École Nationale Supérieure de Chimie de Rennes, CNRS, ISCR (Institut des Sciences Chimiques de Rennes)–UMR 6226, Universite de Rennes, F-35000 Rennes, France

**Keywords:** Ag-NPs/TiO_2_ nanotubes, batch reactor, wastewater treatment, reactive species, kinetic modeling

## Abstract

In this work, we present the influence of the decoration of TiO_2_ nanotubes (TiO_2_-NTs) with Ag silver nanoparticles (Ag-NPs) on the photocatalysis of emerging pollutants such as the antibiotic diclofenac sodium. The Ag-NPs were loaded onto the TiO_2_-NTs by the anodization of metallic titanium foils. Diclofenac sodium is an emerging pollutant target of the pharmaceutical industry because of its negative environmental impact (high toxicity and confirmed carcinogenicity). The obtained Ag-NP/TiO_2_-NT nanocomposites were characterized by X-ray diffraction (XRD), photoluminescence spectroscopy (PL), scanning electron microscopy (SEM), transmission spectroscopy (TEM), and X-ray photoelectron spectroscopy (XPS). In order to study the photocatalytic behavior of Ag-NPs/TiO_2_-NTs with visible cold LEDs, the possible photocatalytic mechanism of antibiotic degradation with reactive species (O_2_°^−^ and OH°) was detailed. Moreover, the Langmuir–Hinshelwood model was used to correlate the experimental results with the optimized catalyst. Likewise, reuse tests showed the chemical stability of the catalyst.

## 1. Introduction

Pharmaceuticals are undoubtedly one of the major advances in modern medicine. Nowadays, they are widely prescribed in humans and animals, both for curative and preventive purposes. However, excessive use can lead to the emergence of resistant bacteria through mechanisms such as (i) enzymatic degradation of antibacterial drugs, (ii) alteration of bacterial proteins that are antimicrobial targets, and (iii) modification in membrane permeability to antibiotics [1,2,3]. In addition, these substances are not completely metabolized by organisms. The immediate consequence is that a certain unmetabolized amount ends up directly in the soil and in surface water [4,5].

Thus, the sources of pharmaceutical substances in the environment are hospital and domestic discharges, which end up in wastewater treatment plants where they are not completely degraded and therefore discharged into surface water. The concentrations in the receiving environment can thus vary according to the biodegradability of the molecules as well as the capacity of the water treatment stations to eliminate or transform them, as these are designed and mainly sized to treat the so-called physicochemical parameters “conventional” (suspended solids, BOD_5_, DCO, nitrogen, phosphorus) [5]. The immediate consequence of such a situation is that for the past fifteen years, drug residues have been found in river water because some treatment plants are still not equipped with effective processes to retain or degrade them [6,7,8,9,10].

To date, several emerging and promising technologies provide a solution to this problem. Among these, the heterogeneous photocatalysis with visible light reveals interesting prospects in terms of degradation/mineralization of compounds, with low energy consumption. Recent studies on model pharmaceutical compounds indicate that this technology has been explored and developed.

In the past decade, titanium dioxide (TiO_2_) has been one of the most studied materials thanks to its unique chemical and physical properties, including its high chemical stability, high resistance to photo corrosion, and its low cost [11]. Nanostructured TiO_2_ has also been used in many applications such as sensors [12], photocatalysis [13,14,15,16], and solar energy conversion [17]. TiO_2_ nanotubes have attracted particular attention because of their high specific surface area [18], high photoactivity [19], and the rapid transfer rate of holes and electron–hole pairs (e^−^/h^+^) photogenerated along the nanotubes [20,21,22]. However, two major factors limit the performance of TiO_2_. On the one hand, due to its wide forbidden energy band (3.2 eV for anatase TiO_2_), the optical absorption of TiO_2_ is limited to the UV spectrum. On the other hand, the photogenerated (e^−^/h^+^) pairs exhibit a high rate of recombination due to a high density of crystal defects [23]. To overcome these problems, many strategies have been established, such as doping TiO_2_ (metallic or non-metallic dopants) [13,17] and coupling with other semiconductors [19,20,24], which could expand absorption under visible light and improve the lifetime of the photogenerated (e^−^/h^+^) pairs. Recently, it has been shown that the decoration of TiO_2_ nanotubes with noble metal nanoparticles can increase their absorption in the visible range, thanks to the surface plasmon resonance (SPR) [25] induced in the metallic nanoparticles in the presence of a light wave. The SPR of the noble metal can be adjusted by controlling the size, shape, and dispersion of the nanoparticles. Nanoparticles of silver (Ag) are used to improve the photoactivity of TiO_2_ nanotubes due to the favorable arrangement of the energy level, which allows the electrons excited by SPR on the Ag nanoparticles to be transferred into the TiO_2_ nanotubes in the presence of visible light. In addition, chemical pathways have been used to decorate TiO_2_ nanotubes with Ag nanoparticles. However, using these techniques cannot control the size and shape of Ag nanoparticles, which can easily accumulate. In addition, the organic agents employed in the growth of Ag nanoparticles can affect the electrical and optical properties of the composite.

In this work, Ag nanoparticles were deposited onto TiO_2_ nanotubes using the photoreduction process, which allows the size of nanoparticles to be controlled by adjusting the UV irradiation time. TiO_2_ nanotubes decorated with Ag nanoparticles were used as photoanodes in a photoelectrochemical system, showing a significant improvement in photo-conversion efficiency. Moreover, nanotubes (TiO_2_-NTs) decorated with Ag silver nanoparticles (Ag-NPs) were used for the photocatalytic degradation of emerging pollutants, such as the antibiotic diclofenac sodium, with visible cold LEDs.

## 2. Setup

The samples were 99.99% purity metal (Ti) plates with a surface area of 2 × 1.5 cm^2^ and a thickness of approximately 0.5 mm. They had identical characteristics to maximize the probability of the same results. In order to achieve surface activation and to avoid any adhesion problems, the samples were first polished using abrasive papers with different grain sizes: 320, 400, 600, 800, 1000, 1200, and 2000. Next, the samples were rinsed with acetone, ethanol, and then bi-distilled water for 10 min in an ultrasonic bath to remove impurities and foreign materials introduced in the polishing step. Finally, the samples were air-dried for some time. Electrochemical experiments were conducted in the laboratory at room temperature. All samples were prepared by anodic oxidation under identical experimental conditions. This was performed in an electrolyte cell containing 100 mL ethylene glycol, 1% ammonium fluoride (NH_4_F), and 2% water (H_2_O). The anodization was 120 min in duration under a fixed voltage of 60 V at a temperature maintained at approximately 25 °C. The samples obtained were calcined for 1 h at 400 °C.

The photoreduction method consists of immersing the TiO_2_ nanotubes in a solution of silver nitrate (AgNO_3_) of 0.1 M concentration for 24 h in the dark to allow the silver ions to be adsorbed on the TiO_2_ surface. Afterward, the samples were rinsed with water and then immersed in methanol under UV illumination (λ = 256 nm) for 10 and 20 min. Under the effect of UV radiation, silver ions (Ag) reduce to metallic form. By increasing the UV irradiation time, Ag nanoparticles increase in size and form agglomerates [1].

**Figure 1:**
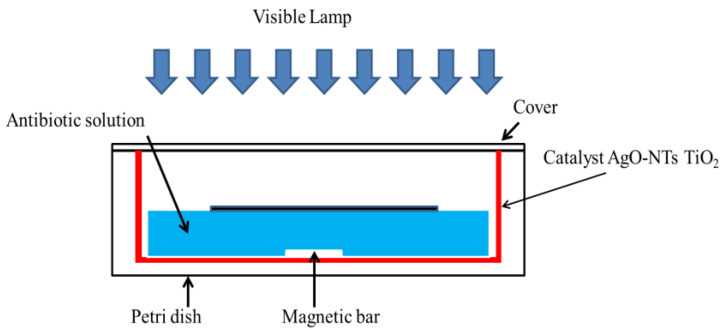
The micro-reactor in a Petri dish used for the treatment of antibiotics by photocatalysis.

The synthesized catalysts were placed in Petri dishes containing the antibiotic solution to be treated. The catalysts were illuminated by visible cold LEDs. This experimental setup is shown in Figure 1. Antibiotic concentration was also monitored by spectrophotochemistry at 270 nm.

Photo-degradation experiments were performed in order to estimate the catalytic efficiencies of TiO_2_-NTs and Ag-NPs/TiO_2_-NTs at various deposition times (10 min, 20 min). The experimental setup consisted of the catalysts (dimensions: 1.2 × 2.5 cm) placed in the photocatalytic batch reactor. An initial antibiotic concentration of 1 mg/L was studied. The lamp was lighted after reaching the adsorption–desorption equilibrium between the catalyst and the antibiotic. For this reason, the reactor containing the assembly was kept in the dark for 1 h before lighting the lamp.

## 3. Results and Discussion

### 3.1. Characterizations of Ag-NPs/TiO_2_-NTs

Scanning electron microscopy (SEM, TESCAN VEGA3) was carried out to visualize the nanostructured morphologies of samples. The elemental analysis of samples was determined by energy-dispersive X-ray spectroscopy (EDS). The TEM and HRTEM images were obtained with the FEI Tecnai G20 microscope operating at 200 kV and equipped with the LaB6 filament. The X-ray photoelectron spectroscopy (XPS) measurements were carried out using a Jeol JPS-9200 photoelectron spectrometer with an achromatic Mg/Al X-ray source at 500 W. The spectra excitations were performed using Mg Kα radiation (1253.6 eV). During XPS data acquisition, the C1s (285.0 eV) peak was used as a reference to correct XPS data from sample charging. The X-ray diffractometer (Cu Ka radiation, λ = 1.5406 Å, PANalytical B.V., Almelo, The Netherlands) was used to identify the crystalline structure and the Pt nanoparticles phase. The photoluminescence (PL) spectra were recorded with a PerkinElmer spectrophotometer equipped with a xenon lamp at an excitation wavelength λ = 340 nm.

#### Scanning Electron Microscopy (SEM)

**Figure 2:**
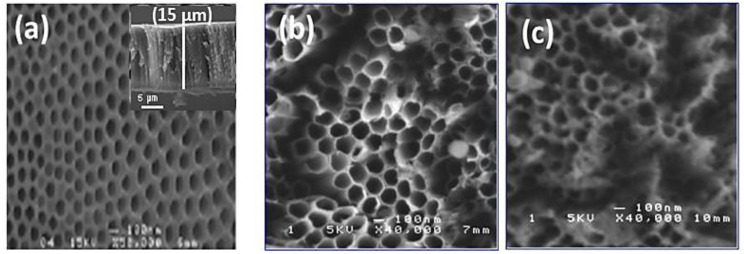
Typical SEM images of TiO_2_-NTs (**a**) before and (**b**) after their Ag-NPs decoration at 10 min and (**c**) 20 min.

Figure 2 shows SEM images of TiO_2_ nanotubes decorated with Ag nanoparticles obtained with 10 min and 20 min pulse durations. The TiO_2_-NTs, without metallic nanoparticles, were vertically aligned on titanium substrates. These nanotubes had a diameter of around 100 nm and a length of around 15 μm (inset Figure 2a). We note the presence of small, uniformly dispersed Ag nanoparticles, and a few aggregates at the top of the nanotubes. The images show a nano-tubular structure with an average diameter of 150 nm. The Ag nanoparticles are very small in size, so they are not observable with SEM.

**Figure 3:**
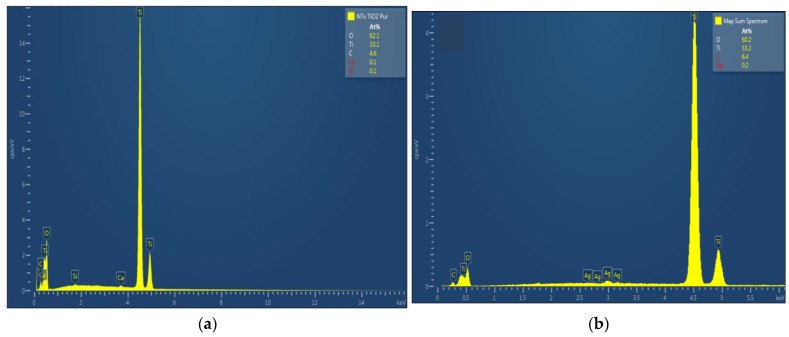

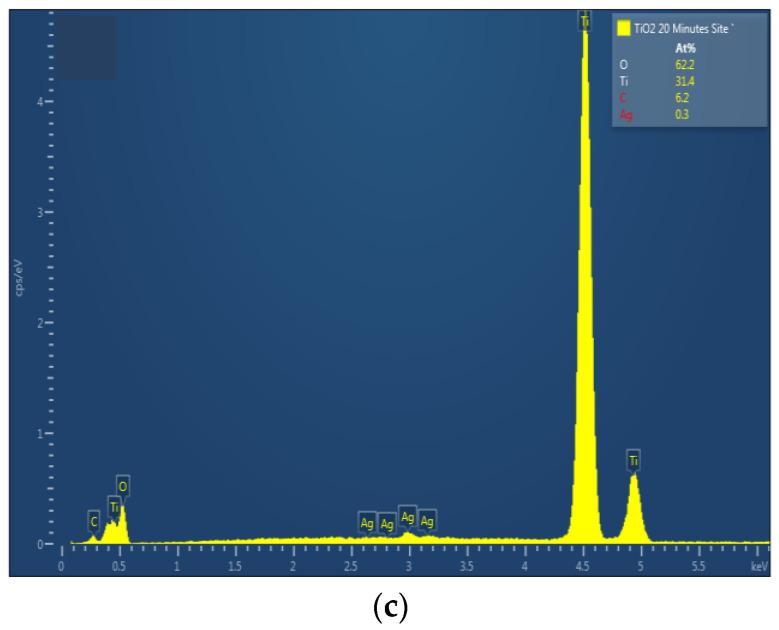
EDX spectra of TiO_2_ nanotubes decorated with silver nanoparticles: (**a**) undecorated TiO_2_-NTs, (**b**) 10 min Ag-NPs/TiO_2_-NTs, and (**c**) 20 min Ag-NPs/TiO_2_-NTs.

The chemical composition of TiO_2_ nanotubes decorated with Ag nanoparticles was estimated by EDX. Figure 3 shows the EDX spectra of TiO_2_ nanotubes decorated with Ag nanoparticles; the atomic percentage of silver increases with photo-reduction time: 0.2 at. % for 10 min Ag/TiO_2_ and 0.3 at. % for 20 min Ag/TiO_2_.

**Figure 4:**
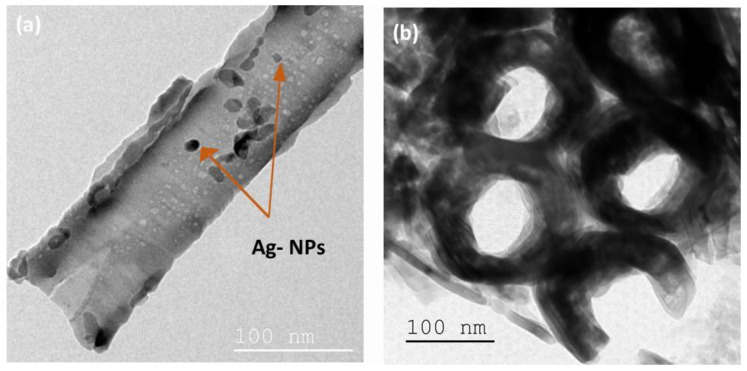
(**a**) Top view and (**b**) cross-section TEM images of Ag-NPs (20 min)/TiO_2_-NTs array.

The TiO_2_-NTs adorned with Ag-NPs were examined by TEM (Figure 4) to verify the presence of Ag-NPs in the TiO_2_-NTs. A drop of Ag-NPs/TiO_2_-NTs dispersed in solution was applied to the TEM holey carbon grids.

Figure 4a reveals the presence of arbitrary shapes of Ag-NPs on the TiO_2_-NTs’ surface.

**Figure 5:**
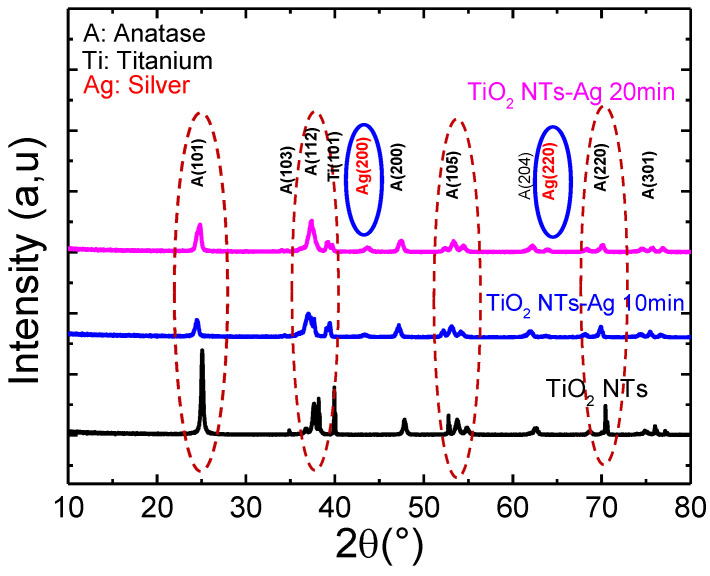
X-ray diffractogram of pure TiO_2_ nanotubes decorated with silver nanoparticles.

Figure 5 shows the X-ray diffractograms of pure TiO_2_ nanotubes decorated with Ag nanoparticles annealed at 400 °C. All samples crystallized in the anatase structure.

Here, we note that the preferential orientation peak (101) characteristic of the anatase phase of titanium oxide continues to decrease with the increase in the deposition time of silver nanoparticles on TiO_2_ nanoparticles. In addition, it is noted that all the samples crystallize in the anatase structure, and the additional peaks characterizing Ag can only be observed from the diffractogram of the TiO_2_-NTs decorated with silver NPs at 20 min; they cannot be observed for the deposition time of less than 10 min. Indeed, below 20 min of UV irradiation, the amount of Ag incorporated is too low to be detected by this characterization technique.

**Figure 6:**
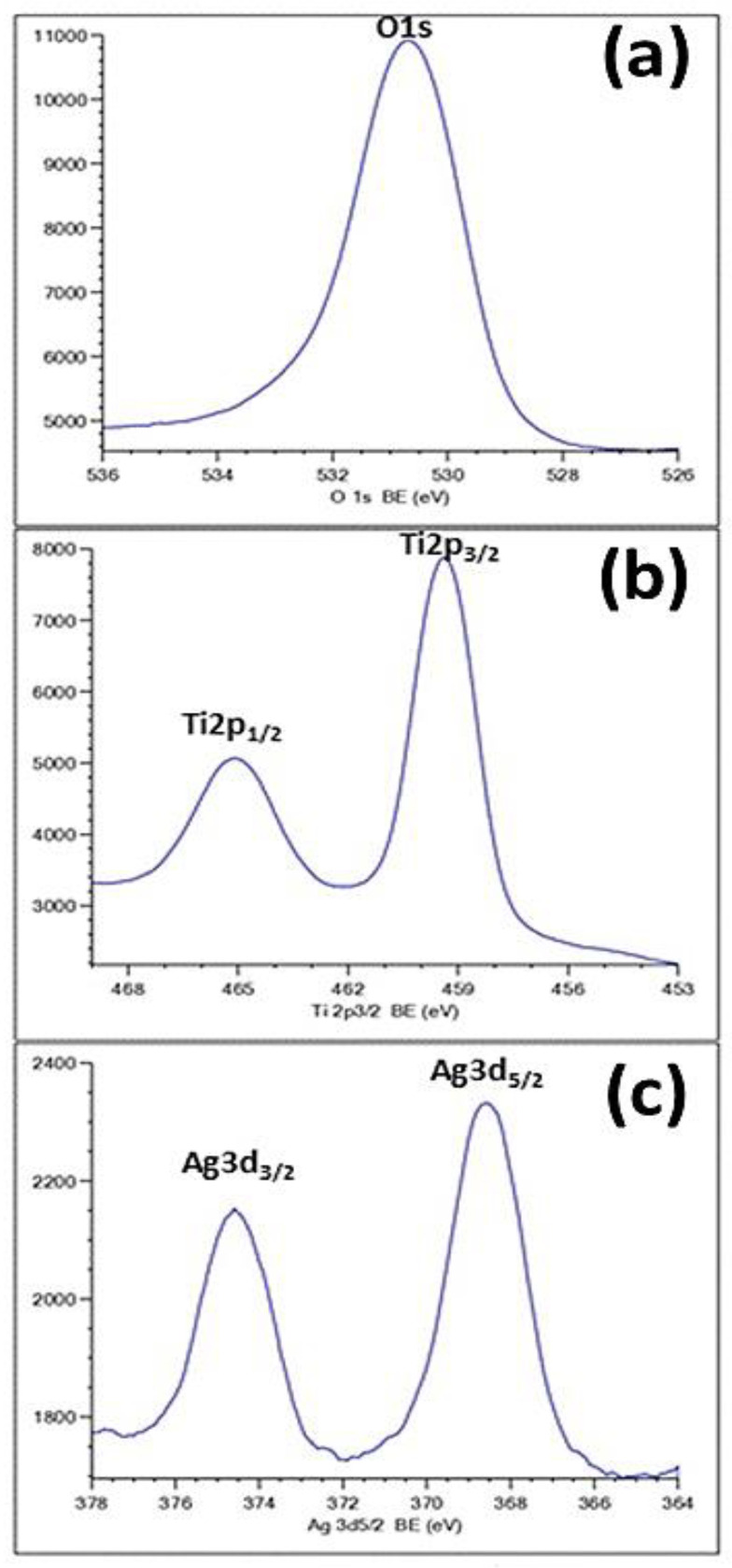
XPS spectra of Ag-NPs (20 min)/TiO_2_-NTs: (**a**) O1s; (**b**) Ti2p; (**c**) Ag 3d.

As can be seen in Figure 6, the formation of Ag species was confirmed by the XPS results. Figure 6a exhibits an O1s peak at 530.8 eV, attributed to the oxide. The appearance of the Ti-2p peak position at a binding energy of 459 eV (Figure 6b) proved that the main chemical valence of Ti is +4. This finding corroborates the assigned values for anatase TiO_2_ in the NIST database. Figure 6c shows the typical XPS signature of the Ag 3d doublet (3d_5/2_ and 3d_3/2_) taken from the surface of the TiO_2_-NTs decorated with Ag-NPs. The signals at 374.3 and 368.3 eV are attributed to Ag 3d_3/2_ and Ag 3d_5/2_ of Ag-NPs, respectively.

These energies often correspond to the metal silver [26], proving the fully metallic character of the used Ag-NPs. This result confirms the effectiveness of the photoreduction process used in the preparation step.

**Figure 7:**
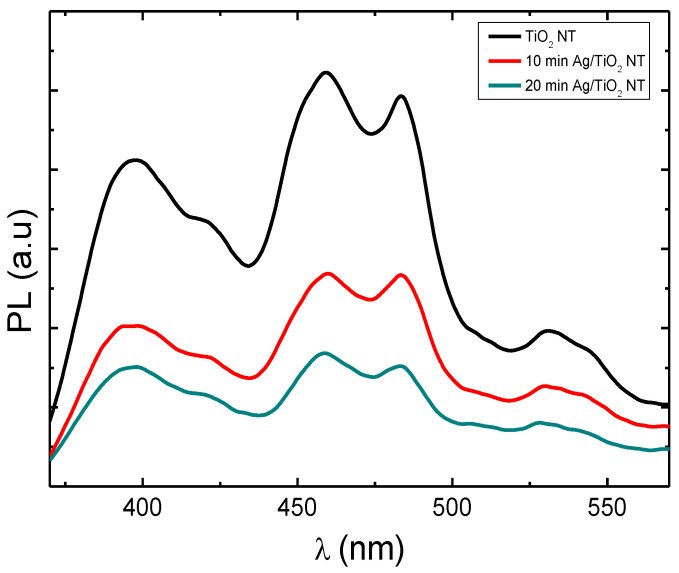
Photoluminescence spectra of pure TiO_2_ nanotubes decorated with silver nanoparticles.

The photoluminescence (PL) of TiO_2_-NTs can inform us about the lifespan and transport of photogenerated charges. Figure 7 shows the photoluminescence spectra of pure TiO_2_-NTs decorated with Ag-NPs. The peak located at 365 nm (3.4 eV) is attributed to the electron transition between the valence and conductive bands of TiO_2_. The peaks present at 459 nm, 483 nm, and 531 nm are attributed to the oxygen vacancies present on the surface of TiO_2_ [27,28].

TiO_2_-NTs decorated with Ag-NPs exhibit a lower PL intensity compared to that of pure TiO_2_, which shows that Ag nanoparticles reduce the density of radiative recombination centers (luminescent centers). This result can be explained by the migration of electrons photogenerated under UV radiation (λ_excitation_ = 340 nm) from the conduction band towards Ag nanoparticles, thus reducing radiative recombination within TiO_2_ [29].

### 3.2. Photocatalytic Experiments 

#### 3.2.1. Effect of Silver Decoration

**Figure 8:**
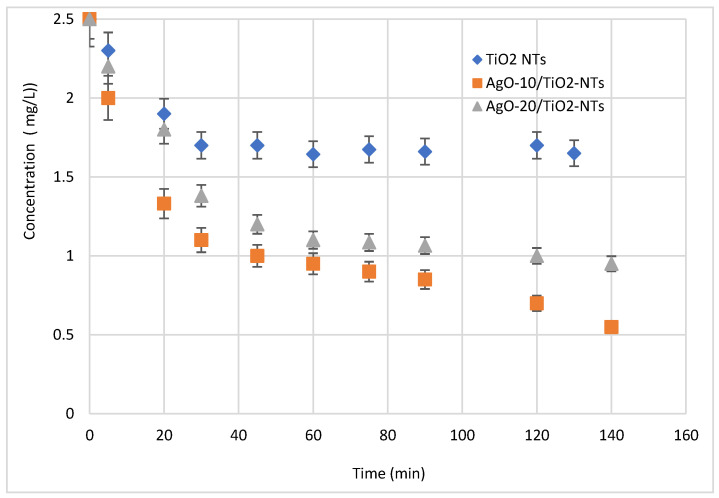
Effect of decoration of Ag-NPs on the photocatalytic activity of the Ag-NP/TiO_2_-NT photocatalyst.

The photocatalytic performances of Ag-NP/TiO_2_-NT catalysts were estimated by photocatalytic degradation of the antibiotic under visible light irradiation at ambient temperature. Figure 8 shows the pollutant removal using Ag-NPs/TiO_2_-NTs decorated by photoreduction for 10 and 20 min. It is seen from Figure 8 that the pollutant removal was pertinently affected by the different decoration times. In this test, it is readily seen that the concentrations of pollutants decreased over time under visible light irradiation. Moreover, a decoration of Ag in 10 min increased the pollutant removal from 35% to 80% of the initial concentration of 2.5 mg/L. This enhancement is related to the availability of the generated active sites at the interface of the catalyst deposited at 10 min [30]. However, the pollutant degradation will tend to be limited at high time values, because these values are too high to form multiple layers of catalysts, thus generating different ions and charges, which leads to distinguished charges at the interfaces of thin films and coverage of all TiO_2_-NTs. 

#### 3.2.2. Effect of Inlet Concentration: Kinetic Modeling 

**Figure 9:**
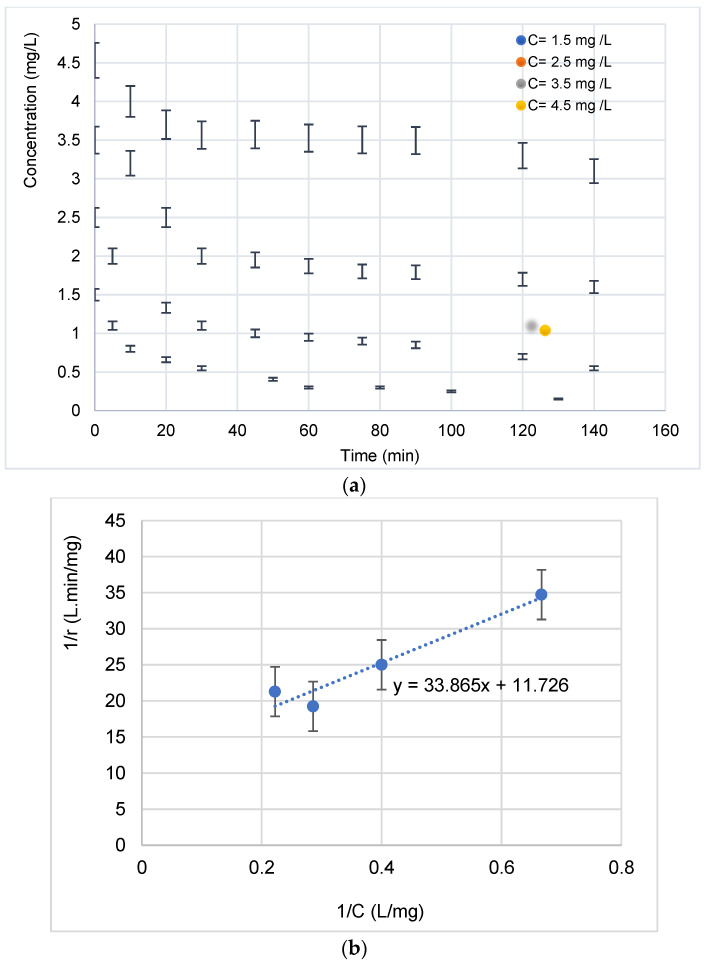
(**a**) Evolution of the concentration of the diclofenac antibiotic during its photocatalytic degradation as a function of time for 4 initial concentrations. (**b**) Linearization of the Langmuir–Hinshelwood isotherm during the degradation of the antibiotic (calculation of k_c_ and K constants).

A representation of the change in concentration over time for four initial concentrations is shown in Figure 9a. As was observed, the increase in the concentration generally favors the pollutant degradation.

At low concentrations, the kinetics are faster [31]. This can be explained by the fact that more molecules are “available” in the solution and that visible light accesses the surface of the catalyst more easily, which leads to an increase in the speed of photocatalytic degradation [32,33,34,35]. However, at high concentrations, molecules begin to act as a filter for incident visible light, so that light hardly reaches the surface of the TiO_2_, resulting in slower photocatalytic degradation [28,36,37,38,39,40]. This same trend has been observed by [41]. Under visible light, electron–hole pairs were produced in the valence bands (VB) of AgO and TiO_2_. Then, the photo-generated electrons passed from the valence bands to their conduction bands (CB). At the same time, electrons can be rapidly transferred to the semiconductor (TiO_2_) interface, which has been reported in Au core–Cu_2_O shell particles [21,25]. This could be due to the fact that the heterojunction in Ag-AgO/TiO_2_ assisted the transfer of photo-generated electrons and holes. Thus, these electrons can quickly be transferred into the TiO_2_ interface and then undergo a transformation, as described below [21].

AgO/TiO_2_ + hʋ → e^−^ + h^+^ (1)

e^−^ + h^+^/AgO → e^−^/Ag → e^−^/TiO_2_ (2)

O_2_ + e^−^ → O_2_.^−^ (3)

O_2_.^−^ + 2H_2_O + 2e^−^ → 3OH^−^ + OH^∙^ (4)

H^+^ + H_2_O → H^+^ + OH^∙^ (5)

Antibiotic+ O_2_.^−^/OH^∙^ → CO_2_ + H_2_O + other products (6)

To describe the Ag (10 min)/TiO_2-_NTs’ catalytic performance, the Langmuir–Hinshelwood (L-H) model was used [42]:

(7) $$r_{0}=-\frac{d\left[pollutant\right]}{dt}=k_{c}\frac{K{\left[pollutant\right]}_{0}}{1+K{\left[pollutant\right]}_{0}}$$

where *r_0_* (mg·L^−1^ min^−1^) is the initial photodegradation rate, [*pollutant*] is the initial BUT concentration (mg/L), *K* is the adsorption constant (L/mg), and *k_c_* is the kinetic constant (mg·L^−1^ min^−1^) at maximum coverage of the experimental conditions. 

The plot of 1/*r_0_* versus 1/[*pollutant*]_0_ (Figure 9b) allows determining *k_c_* and *K* values. The linearized (L-H) equation is:

(8) $$\frac{1}{r_{0}}=\frac{1}{k_{c}K}\times \frac{1}{{\left[pollutant\right]}_{0}}+\frac{1}{k_{C}}$$

Table 1 illustrates the kinetic and adsorption constants of L-H. 

### 3.3. Catalyst Reusability

Catalyst reusability is a major issue to look into because it measures how well a catalyst can degrade contaminants in solution. Therefore, several visible photocatalytic experiments were performed to examine the ability of the elaborated silver sample to degrade antibiotics in four successive cycles. 

**Figure 10:**
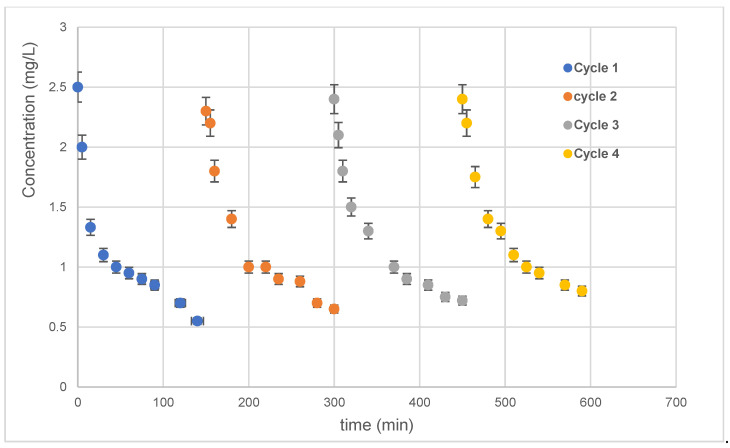
Four successive cycles of photocatalytic oxidations using the 10 min Ag-NP/TiO_2_-NT catalyst.

After each degradation cycle, the 10 min Ag-NPs/TiO_2_-NTs were well washed and reused. The photocatalytic findings of repetitive use (four cycles) of the 10 min Ag-NP/TiO_2_-NT photocatalyst are shown in Figure 10. Figure 10 shows no loss of sample photoactivity. These results prove the overall potential of the 10 min Ag-NP/TiO_2_-NT catalyst for water treatment applications. Generally, the same slope is noted in each cycle. Consequently, these results shown below prove the availability of active sites and the excellent photocatalytic stability of this catalyst.

## 4. Conclusions

The titanium electrochemical anodization and the photochemical reduction of Ag-NPs on TiO_2_-NTs were successfully used to create the Ag-NP/TiO_2_-NT nanocomposites. Homogeneous nanotubes with a diameter of 100 nm were used to synthesize TiO_2_-NTs, which were then crystallized in the anatase phase. The Ag-NP deposition was performed at various electrodeposition times. The Ag-NPs’ adjunction to the TiO_2_-NTs increased the visible light absorption until almost 500 nm. Antibiotic removal experiments were conducted by TiO_2_-NTs and Ag-NPs/TiO_2_-NTs with various photoreduction times under visible light (380–720 nm). These tests revealed a high photocatalytic efficiency. This finding can be explained by the combination of the energy band level positions of semiconductors and the high visible light absorption of Ag nanoparticles. The Ag-NP/TiO_2_-NT photocatalytic system significantly improved the antibiotic degradation concerning the TiO_2_-NTs alone, because of the extended photoresponse and the efficient photogeneration of electron–hole pairs under UV/visible light. Thus, this work shows the high stability of the Ag-NP/TiO_2_-NT nanocomposites under UV light irradiation.

## Figures and Tables

**Table 1:** L-H constants (k_c_ and K) on the 10 min AgO-NPs/TiO_2_-NT catalyst.

k_c_: Kinetic Constant of L-H (mg/(L. min))	K: Adsorption Constant of L-H (L/mg)
2.9 × 10^−3^	2.88

## Data Availability

Not applicable.
